# Bioinformatic analysis of the gene expression profile in muscle atrophy after spinal cord injury

**DOI:** 10.1038/s41598-021-01302-6

**Published:** 2021-11-09

**Authors:** Hui Huang, Jinju Xue, Jiaxuan Zheng, Haiquan Tian, Yehan Fang, Wei Wang, Guangji Wang, Dan Hou, Jianping Lin

**Affiliations:** 1 Department of Sports Medicine, Hainan General Hospital (Hainan Affiliated Hospital of Hainan Medical University), Haikou, 570311 Hainan China; 2 Department of Geriatrics, Affiliated Haikou Hospital, Central South University Xiangya School of Medicine, Haikou, 570208 Hainan China; 3 Department of Pathology, Hainan General Hospital (Hainan Affiliated Hospital of Hainan Medical University), Haikou, 570311 Hainan China; 4 Department of Orthopaedic Surgery, The Second People’s Hospital of Changzhi, Changzhi, 046000 Shanxi China; 5 Department of Emergency, Hainan General Hospital (Hainan Affiliated Hospital of Hainan Medical University), Haikou, 570311 Hainan China; 6 Department of Neurology, Affiliated Haikou Hospital, Central South University Xiangya School of Medicine, Haikou, 570208 Hainan China; 7 Department of Joint Surgery, Hainan General Hospital (Hainan Affiliated Hospital of Hainan Medical University), Haikou, 570311 Hainan China

**Keywords:** Biological techniques, Genetics

## Abstract

Spinal cord injury (SCI) is often accompanied by muscle atrophy; however, its underlying mechanisms remain unclear. Here, the molecular mechanisms of muscle atrophy following SCI were investigated. The GSE45550 gene expression profile of control (before SCI) and experimental (14 days following SCI) groups, consisting of Sprague–Dawley rat soleus muscle (n = 6 per group), was downloaded from the Gene Expression Omnibus database, and then differentially expressed gene (DEG) identification and Gene Ontology, pathway, pathway network, and gene signal network analyses were performed. A total of 925 differentially expressed genes, 149 biological processes, and 55 pathways were screened. In the pathway network analysis, the 10 most important pathways were citrate cycle (TCA cycle), pyruvate metabolism, MAPK signalling pathway, fatty acid degradation, propanoate metabolism, apoptosis, focal adhesion, synthesis and degradation of ketone bodies, Wnt signalling, and cancer pathways. In the gene signal network analysis, the 10 most important genes were *Acat1*, *Acadvl*, *Acaa2*, *Hadhb*, *Acss1*, *Oxct1*, *Hadha, Hadh*, *Acaca*, and *Cpt1b*. Thus, we screened the key genes and pathways that may be involved in muscle atrophy after SCI and provided support for finding valuable markers for this disease.

## Introduction

Spinal cord injury (SCI) is a major cause of disability in humans which can lead to muscle atrophy. Approximately 20–55% of muscle atrophy cases appear within 6 months to 1 year after complete SCI^1^; 20–30% of muscle atrophy cases appear within 6 months to 1 year after incomplete SCI^2^. Muscle atrophy not only affects the care management and activities of daily living for patients with SCI, but also has significant effects on their health by increasing the risk of secondary complications, such as osteoporosis, diabetes, and cardiovascular disease^3–6^. Thus, prevention of muscle atrophy is essential for maintaining metabolic health and normal life activities after SCI^7^.

Previous studies^8–10^ have shown that the changes in pathways associated with protein ubiquitination and energy generation are shared features in the process of muscle atrophy. Growth factors such as insulin-like growth factor^11^, myogenic regulatory factors^12^, transforming growth factors^13^, and bone morphogenetic proteins^14^ may play important roles in muscle atrophy. However, the aetiology and precise underlying mechanisms of muscle atrophy following SCI are still poorly understood. To date, only one global gene profiling study on the changes in gene expression in muscle tissue in the first few days (3 and 5 days) after SCI has been conducted; studies at later timepoints are lacking. The GSE45550 microarray dataset containing gene chip data of Sprague–Dawley rat soleus muscle tissue before SCI and 14 days after SCI was generated in a study by Baligand et al.^7^; however, an in-depth bioinformatics analysis was not conducted.

In the present study, we downloaded the GSE45550 gene profile dataset and conducted an in-depth bioinformatic analysis of gene profile data before and 14 days after SCI to obtain relevant genes, biological processes, and pathways that may play an important role in the development of muscle atrophy after SCI. We aimed to expand our systematic understanding of the mechanisms of muscle atrophy after SCI.

## Results

### Identification of differentially expressed genes (DEGs)

**Figure 1:**
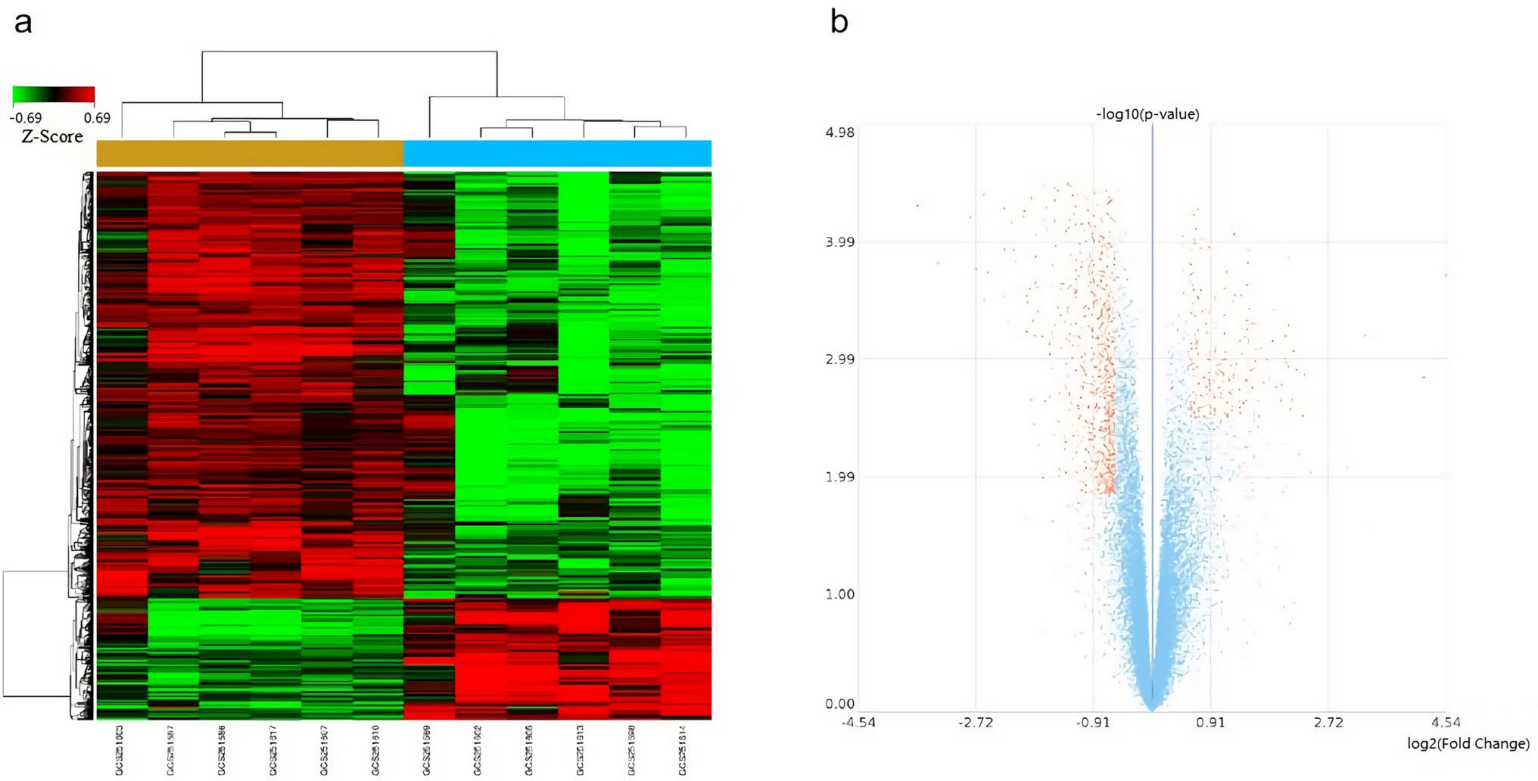
(**a**) Dendrogram of the comparison between the control (before SCI, n = 6/group) and experimental groups (14 days after SCI, n = 6/ group). The X-axis shows the sample names of the two groups, and the Y-axis shows the 925 differentially expressed genes. Red indicates high expression of the differential gene in the grouped samples, whereas green indicates low expression. Above the plot, the yellow strip indicates the control group and blue strip indicates the experimental group. SCI, spinal cord injury. (**b**) Volcano plot of the control (before SCI, n = 6/group) and experimental groups (14 days after SCI, n = 6/group). Orange dots indicate the 925 differentially expressed genes, of which the 720 downregulated genes are to the left and the 205 upregulated genes are to the right of the midline. SCI, spinal cord injury.

After the data was acquired, it was quantiles normalization for data analysis. Compared with those in the control group (before SCI, n = 6/group), 925 genes in the experimental group (14 days after SCI, n = 6/group) were significantly differentially expressed in skeletal muscle samples. Of these, 205 genes were upregulated and 720 genes were downregulated. The results are shown as a dendrogram (Fig. 1a) and a volcano plot (Fig. 1b). The 10 genes with the most significant differential expression were *Kcnj11*, *Chmp4c*, *Opn4*, *Ppp3cb*, *Tasp1*, *Gsr*, *Stk40*, *Alpk3*, *Pla2g12a*, and *Atf5* (Table 1), all of which were downregulated.

**Table 1:** Top 10 differentially expressed genes (in order of increasing *p*-value).

Gene symbol	Gene description	d score	Fold change	*p* value	q-value	Gene Feature	Rank
*Kcnj11*	Potassium inwardly rectifying channel, subfamily J, member 11	− 11.28581	− 2.47459	3.30E−05	< 0.001	Down	1
*Chmp4c*	Charged multivesicular body protein 4C	− 10.755534	− 2.476554	3.40E−05	< 0.001	Down	2
*Opn4*	Opsin 4	− 9.236353	− 2.399959	3.50E−05	< 0.001	Down	3
*Ppp3cb*	Protein phosphatase 3, catalytic subunit, beta isozyme	− 9.232755	− 2.854103	3.60E−05	< 0.001	Down	4
*Tasp1*	Taspase, threonine aspartase 1	− 8.215108	− 2.756438	3.80E−05	< 0.001	Down	5
*Gsr*	Glutathione reductase	− 8.231781	− 1.642069	3.80E−05	< 0.001	Down	6
*Stk40*	Serine/threonine kinase 40	− 8.140561	− 1.749477	3.90E−05	< 0.001	Down	7
*Alpk3*	Alpha-kinase 3	− 7.866514	− 1.846566	4.00E−05	< 0.001	Down	8
*Pla2g12a*	Phospholipase A2, group XIIA	− 7.817663	− 2.232567	4.10E−05	< 0.001	Down	9
*Atf5*	activating transcription factor 5	− 7.712616	− 2.339008	4.20E−05	< 0.001	Down	10

### Gene ontology (GO) analysis

A total of 149 GO terms (biological processes) with statistically significant enrichment were obtained (*p* < 0.05, false discovery rate (FDR) < 0.05). The top 10 GO terms with significant enrichment were ‘response to drug’, ‘fatty acid beta-oxidation’, ‘response to hypoxia’, ‘biological_process’, ‘heart development’, ‘positive regulation of transcription, DNA-dependent’, ‘response to glucose stimulus’, ‘response to nutrient’, ‘positive regulation of transcription from RNA polymerase II promoter’, and ‘positive regulation of apoptotic process’ (Table 2).

**Table 2:** Top 10 gene ontology (GO) terms with statistically significant enrichment (in the order of increasing *p*-value).

GO ID	GO term	*p* value	FDR	Gene symbols	Rank
GO:0,042,493	Response to drug	8.56E−16	1.51E−12	|*Stat1*|*Cdk1*|*Ccnb1*|*Ddit3*|*Kcnj11*|*Lox*|*Vegfb*|*Srebf1*|*Lgals1*|*Nppc*|*Emx2*|*Bdh1*|*Hadha*|*Casp3*|*Tspo*|*Adipoq*|*Abcc8*|*Fabp3*|*Acsl1*|*Hadh*|*Fabp4*|*Timp4*|*Txnrd2*|*Acaca*|*Oxct1*|*Acot2*|*Lpl*|*Pparg*|*Mdk*|*Gnas*|*Ak4*|*Aqp7*|*Gnpat*|*Ldha*|*Cd36*|	1
GO:0,006,635	Fatty acid beta-oxidation	1.02E−13	8.97E−11	|*Echs1*|*Acaa2*|*Fabp3*|*Acadvl*|*Acox2*|*Adipoq*|*Hadha*|*Ech1*|*Cpt1b*|*Hadh*|*Eci1*|*Hadhb*|	2
GO:0,001,666	Response to hypoxia	5.44E−11	3.20E−08	|*Ddit4*|*Cat*|*Adrb2*|*Ppara*|*Pak1*|*Nppc*|*Mmp14*|*Kcnma1*|*Casp3*|*Sox4*|*Plod2*|*Acot2*|*Pdlim1*|*Ldha*|*Egln1*|*Myocd*|*Adipoq*|*Tfrc*|*Narfl*|*Cxcl12*|*Loxl2*|	3
GO:0,008,150	Biological_process	8.71E−11	3.85E−08	|*Gstm5*|*Ldb3*|*Tmem106b*|*Myoc*|*Ablim1*|*Smyd2*|*Impa2*|*Abca8a*|*Xpo4*|*Hccs*|*Cdkn3*|*Adam19*|*Mrpl40*|*Clmp*|*Gca*|*Trim14*|*Cks2*|*Ywhah*|*Baiap2l1*|*Col5a1*|*Trim16*|*Tasp1*|*Mkks*|*Cbfb*|*LOC306766*|*Xpa*|*Scn4b*|*Abhd1*|*Trim35*|*Pbx1*|*Wnt9a*|*Kif21a*|*Rpusd4*|*Adhfe1*|*Ino80c*|*Mllt11*|*Tmem120a*|*Tmem127*|*Sorbs1*|*Opa3*|*Wnt16*|*Nrtn*|*Pcbd1*|*Kdm8*|*Gcat*|*Eya1*|*Ndufs1*|*Ndufa8*|*Col6a1*|*Clasp1*|*Cul2*|*Large*|*Pex19*|	4
GO:0,007,507	Heart development	3.69E−10	1.31E−07	|*Adam19*|*Ppp3cb*|*Ppara*|*Txnrd2*|*Col3a1*|*Kcnj8*|*Myocd*|*Alpk3*|*Pparg*|*Rxrg*|*Myl2*|*Vegfb*|*Casp3*|*Cxadr*|*Sox4*|*Pkp2*|*Rps6ka2*|*Oxct1*|	5
GO:0,045,893	positive regulation of transcription, DNA-dependent	7.37E−10	2.17E−07	|*Stat1*|*Dnajc2*|*Tasp1*|*Patz1*|*Pparg*|*Myocd*|*Mlxipl*|*Mdk*|*Ywhah*|*Klf2*|*Naa15*|*Tp63*|*Kdm8*|*Ppargc1b*|*Srebf1*|*Scx*|*Trim16*|*Ppara*|*Mstn*|*Sox4*|*Pcbd1*|*Eya1*|*Myf6*|*Esrra*|*Lrp5*|*Mllt11*|*Trim24*|*Ddit3*|	6
GO:0,009,749	Response to glucose stimulus	1.64E−09	4.13E−07	|*Nr0b2*|*Col6a2*|*Ldha*|*Glul*|*Nnat*|*Mlxipl*|*Col6a3*|*Ndufa9*|*Ctsl1*|*Irs2*|*Casp3*|*Srebf1*|*Adipoq*|	7
GO:0,007,584	Response to nutrient	4.09E−09	9.03E−07	|*Oxct1*|*Cd36*|*Ldha*|*Stat1*|*Mapt*|*Bdh1*|*Acsl4*|*Nqo1*|*Gnpat*|*Adipoq*|*Ddit3*|*Tfrc*|*A2m*|*Acsl1*|	8
GO:0,045,944	positive regulation of transcription from RNA polymerase II promoter	1.32E−08	2.60E−06	|*Lum*|*Mavs*|*Bex1*|*Cytl1*|*Scx*|*Pbx1*|*Klf2*|*Ablim1*|*Cbfa2t3*|*Sox4*|*Cbfb*|*Vgll2*|*Thrb*|*Esrra*|*Adrb2*|*Ppargc1b*|*Tp63*|*Hoxa5*|*Ddit3*|*Eya1*|*Myocd*|*Bmp6*|*Nfix*|*Cd28*|*Srebf1*|*Stat1*|*Crem*|*Pparg*|*Rxrg*|*Mlxipl*|*Ppara*|*Lrp5*|	9
GO:0,043,065	Positive regulation of apoptotic process	4.44E−08	7.67E−06	|*Dffa*|*Kcnma1*|*Tspo*|*Bid*|*G0s2*|*Casp3*|*Adamtsl4*|*Sfrp4*|*Rps6ka2*|*Srpx*|*Mllt11*|*Sox4*|*Jak2*|*Adrb2*|*Tsc22d1*|*Ldha*|*Ddit3*|*Trim35*|	10

### Pathway analysis

There were 55 KEGG pathways with significant enrichment of DEG (*p* < 0.05). The top 10 KEGG pathways with significant enrichment were ‘metabolic pathways’, ‘PPAR signalling pathway’, ‘peroxisome’, ‘fatty acid degradation’, ‘fatty acid elongation’, ‘glutathione metabolism’, ‘propanoate metabolism’, ‘valine, leucine and isoleucine degradation’, ‘adipocytokine signalling pathway’, and ‘glyoxylate and dicarboxylate metabolism’ (Table 3).

**Table 3:** Top 10 pathways with significant enrichment of differentially expressed genes (in order of increasing *p*-value).

Pathway ID	Pathway name	*P* value	FDR	Gene symbols	Rank
1100	Metabolic pathways	2.56E−17	5.19E−15	|*Ndufv3*|*Acaca*|*Mthfd2l*|*Acot1*|*Nnt*|*Acox2*|*Flad1*|*Idh1*|*LOC100359539*|*LOC691853*|*Fahd1*|*Acsl1*|*Hadh*|*St3gal6*|*Acadvl*|*Cat*|*Mdh1*|*Mgll*|*Ndufs1*|*Pmvk*|*Ak4*|*Acat1*|*Ldha*|*LOC497978*|*Acsl4*|*Coq5*|*Bdh1*|*Synj2*|*Pgp*|*Cda*|*Acot7*|*Impa2*|*Odc1*|*Pccb*|*Hyal3*|*Ugp2*|*Inpp5a*|*Acot2*|*Hpd*|*Alox15*|*Echs1*|*Pank1*|*Mlycd*|*Hadha*|*Pola2*|*Agpat2*|*Pla2g12a*|*Chkb*|*Ndufa9*|*Idh2*|*Pla2g4b*|*Acaa2*|*Pigo*|*Glul*|*Ppcs*|*Pla2g16*|*Acss1*|*Hadhb*|*Extl1*|*Ndufa8|*	1
3320	PPAR signalling pathway	3.29E−12	3.34E−10	|*Sorbs1*|*Fabp3*|*Fabp4*|*Cd36*|*Acox2*|*Pparg*|*Aqp7*|*Ppara*|*Acsl1*|*Rxrg*|*Adipoq*|*Cpt1b*|*Acsl4*|*Lpl*|	2
4146	Peroxisome	8.01E−12	5.42E−10	|*Acsl4*|*Idh1*|*Cat*|*Mlycd*|*Ech1*|*Gstk1*|*LOC100362572*|*Acox2*|*Pmvk*|*Acsl1*|*Idh2*|*Pxmp2*|*Pex19*|*Gnpat*|	3
71	Fatty acid degradation	2.93E−11	1.49E−09	|*Acadvl*|*Cpt1b*|*Eci1*|*Acat1*|*Echs1*|*Acsl1*|*Acsl4*|*Hadhb*|*Hadh*|*Hadha*|*Acaa2*|	4
62	Fatty acid elongation	2.81E−09	1.14E−07	|*Acot7*|*Acot1*|*Hadha*|*Hadh*|*Hadhb*|*Acot2*|*Acaa2*|*Echs1*|	5
480	Glutathione metabolism	7.73E−09	2.61E−07	|*Mgst3*|*Gsr*|*LOC100359539*|*Gstk1*|*Idh1*|*Odc1*|*Gstm5*|*Gsta3*|*Ggct*|*Idh2*|	6
640	Propanoate metabolism	1.64E−08	4.76E−07	|*Acss1*|*Mlycd*|*Echs1*|*Ldha*|*Acaca*|*Pccb*|*Hadha*|*Acat1*|	7
280	Valine, leucine, and isoleucine degradation	7.16E−07	1.82E−05	|*Pccb*|*Oxct1*|*Hadhb*|*Hadh*|*Acat1*|*Hadha*|*Acaa2*|*Echs1*|	8
4920	Adipocytokine signalling pathway	9.37E−07	2.11E−05	|*Cpt1b*|*Adipoq*|*Jak2*|*Irs2*|*Ppara*|*Cd36*|*Rxrg*|*Acsl4*|*Acsl1*|	9
630	Glyoxylate and dicarboxylate metabolism	1.05E-06	2.13E-05	|*Glul*|*Pgp*|*Mdh1*|*Cat*|*Acat1*|*Pccb*|	10

### Pathway network analysis

**Figure 2:**
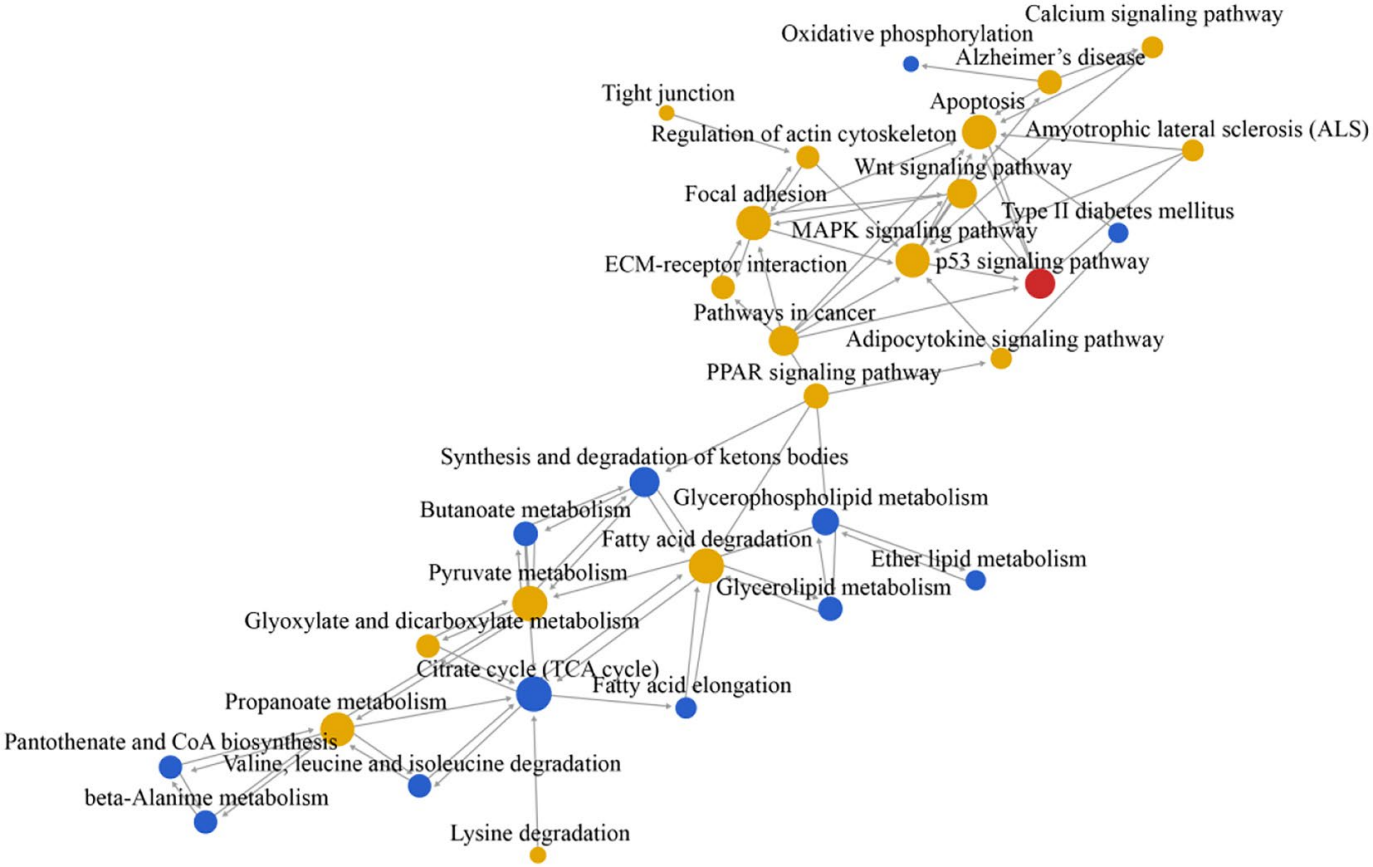
Network diagram of the interaction between the signalling pathways in the KEGG database based on their upstream and downstream relationships. Red dots indicate significant signalling pathways in which upregulated differentially expressed genes were involved, blue dots indicate the significant signalling pathway in which downregulated differentially expressed genes were involved, and yellow dots indicate significant signalling pathways in which both upregulated and downregulated differentially expressed genes were involved. Solid arrows indicate the upstream and downstream relationships between two signalling pathways, with the upstream signalling pathway at the start of the arrow and the downstream signalling pathway at the end of the arrow. Larger points indicate signalling pathways with a greater number of upstream and downstream effects (higher degree) and thus greater importance in the network.

Thirty-one core pathways were identified in pathway network analysis, including 12 downregulated pathways, 1 upregulated pathway, and 18 upregulated/downregulated pathways (Table 4). Figure 2 shows the relationship network of these pathways. Among them, ‘citrate cycle (TCA cycle)’, ‘pyruvate metabolism’, ‘MAPK signalling pathway’, ‘fatty acid degradation’, ‘propanoate metabolism’, ‘apoptosis’, ‘focal adhesion’, ‘synthesis and degradation of ketone bodies’, ‘Wnt signalling pathway’, and ‘pathways in cancer’ are the 10 pathways with the highest degree values, indicating that they are the most important pathways in the network. The larger the degree value, the more the number of signalling pathways with upstream and downstream effects, and the more important they are in the network.

**Table 4:** Core pathways identified from pathway network analysis (in order of decreasing degree-value).

Pathway ID	Pathway name	Outdegree	Indegree	Degree	Pathway feature	Rank
20	Citrate cycle (TCA cycle)	4	7	11	Down	1
620	Pyruvate metabolism	5	5	10	Down|up	2
4010	MAPK signalling pathway	3	7	10	Up|down	3
71	Fatty acid degradation	4	5	9	Down|up	4
640	Propanoate metabolism	5	4	9	Down|up	5
4210	Apoptosis	1	8	9	Up|down	6
4510	Focal adhesion	5	4	9	Up|down	7
72	Synthesis and degradation of ketone bodies	3	4	7	Down	8
4310	Wnt signalling pathway	4	3	7	Down|up	9
5200	Pathways in cancer	7	0	7	Up|down	10
564	Glycerophospholipid metabolism	3	3	6	Down	11
4115	p53 signalling pathway	1	5	6	Up	12
650	Butanoate metabolism	3	2	5	Down	13
3320	PPAR signalling pathway	4	1	5	Down|up	14
280	Valine, leucine, and isoleucine degradation	2	2	4	Down	15
410	beta-Alanine metabolism	2	2	4	Down	16
561	Glycerolipid metabolism	2	2	4	Down	17
630	Glyoxylate and dicarboxylate metabolism	2	2	4	Down|up	18
770	Pantothenate and CoA biosynthesis	2	2	4	Down	19
4810	Regulation of actin cytoskeleton	2	2	4	Up|down	20
5010	Alzheimer's disease	3	1	4	Up|down	21
62	Fatty acid elongation	1	2	3	Down	22
4020	Calcium signalling pathway	2	1	3	Down|up	23
4512	ECM-receptor interaction	1	2	3	Down|up	24
4920	Adipocytokine signalling pathway	1	2	3	Down|up	25
5014	Amyotrophic lateral sclerosis (ALS)	3	0	3	Up|down	26
565	Ether lipid metabolism	1	1	2	Down	27
4930	Type II diabetes mellitus	2	0	2	Down	28
310	Lysine degradation	1	0	1	Down|up	29
4530	Tight junction	1	0	1	Up|down	30
190	Oxidative phosphorylation	0	1	1	Down	31

### Gene signal network analysis

**Figure 3:**
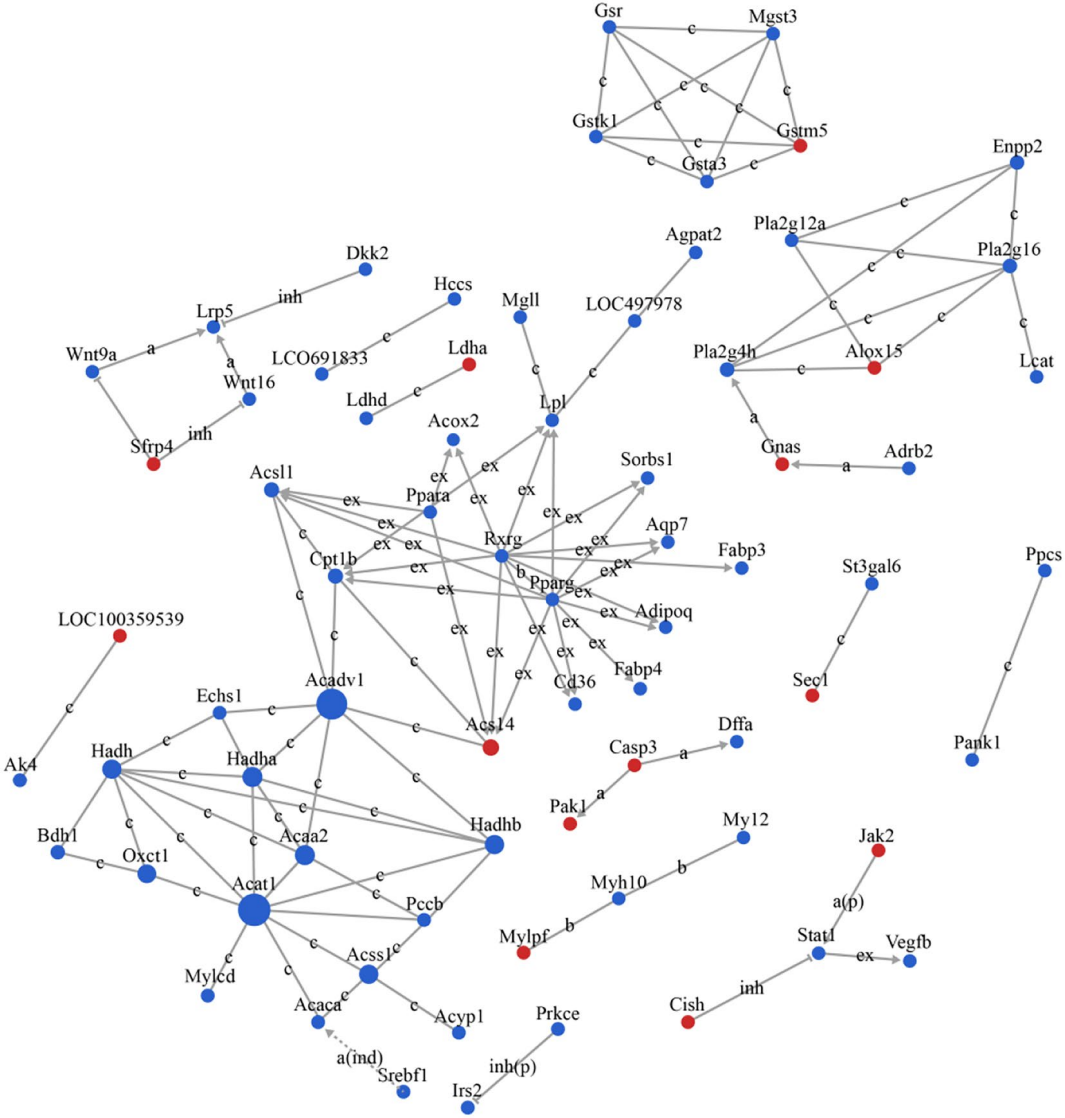
Network diagram of the relationship between 72 hub genes selected from gene signal network analysis. Each dot in the figure represents a gene. Larger points indicate greater betweenness values and greater importance. Red dots represent upregulated genes and blue dots represent downregulated genes. Lines indicate interaction between two genes, and the labels are the abbreviations of the type of interaction between the two genes (compound: c, active: a, indirect: ind, inhibition: inh, expression: ex, binding: b). Solid lines without arrows indicate non-directional interaction. Solid arrows indicate directional interaction, with the upstream gene at the start of the arrow and the downstream gene at the end of the arrow. Dotted arrows indicate directional interaction, with the upstream gene at the start of the arrow and the downstream gene at the end of the arrow, but the relationship between the two is indirect. Solid lines with flat heads indicate directional interaction, with the upstream gene at the start and the downstream gene at the end, with the upstream gene having an inhibitory effect on the downstream gene.

**Figure 4:**
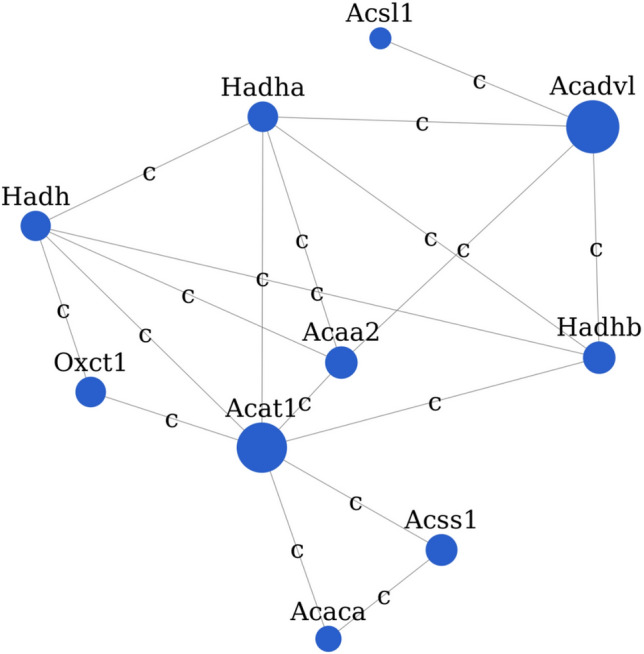
Network diagram of the hub genes *Acat1*, *Acadvl*, *Acaa2*, *Hadhb*, *Acss1*, *Oxct1*, *Hadha*, *Hadh*, *Acaca*, and *Cpt1b*. Each dot in the figure represents a gene. Larger points indicate greater betweenness values. Blue dots represent downregulated genes. Lines indicate interaction between two genes, and the labels are the abbreviations of the type of interaction between the two genes (compound: c). Solid lines without arrows indicate non-directional interaction.

Seventy-two hub genes were identified in the gene signal network analysis, of which 13 were upregulated and 59 were downregulated. Figure 3 shows the relationship network of these genes. The 10 hub genes with the highest betweenness values (higher values indicate greater importance) were *Acat1, Acadvl, Acaa2, Hadhb, Acss1, Oxct1, Hadha, Hadh, Acaca,* and *Cpt1b* (Table 5). The network map of these genes is shown in Fig. 4.

**Table 5:** Top 10 hub genes from gene signal network analysis based on betweenness (in order of decreasing betweenness-value).

Gene symbol	Gene feature	Gene description	Betweenness	Indegree	Outdegree	Degree	Rank
*Acat1*	Down	Acetyl-CoA acetyltransferase 1	125.4286	8	8	16	1
*Acadvl*	Down	Acyl-CoA dehydrogenase, very long chain	123.4	7	7	14	2
*Acaa2*	Down	Acetyl-CoA acyltransferase 2	41.6024	5	5	10	3
*Hadhb*	Down	Hydroxyacyl-CoA dehydrogenase/3-ketoacyl-CoA thiolase/enoyl-CoA hydratase (trifunctional protein), beta subunit	41.6024	5	5	10	4
*Acss1*	Down	Acyl-CoA synthetase short-chain family member 1	39.1333	4	4	8	5
*Oxct1*	Down	3-Oxoacid CoA transferase 1	34	3	3	6	6
*Hadha*	Down	Hydroxyacyl-CoA dehydrogenase/3-ketoacyl-CoA thiolase/enoyl-CoA hydratase (trifunctional protein), alpha subunit	33.669	6	6	12	7
*Hadh*	Down	Hydroxyacyl-CoA dehydrogenase	32.0714	6	6	12	8
*Acaca*	Down	Acetyl-CoA carboxylase alpha	16	3	2	5	9
*Cpt1b*	Down	Carnitine palmitoyltransferase 1b, muscle	15	6	3	9	10

### Results of quantitative real time PCR (qRT-PCR)

**Figure 5:**
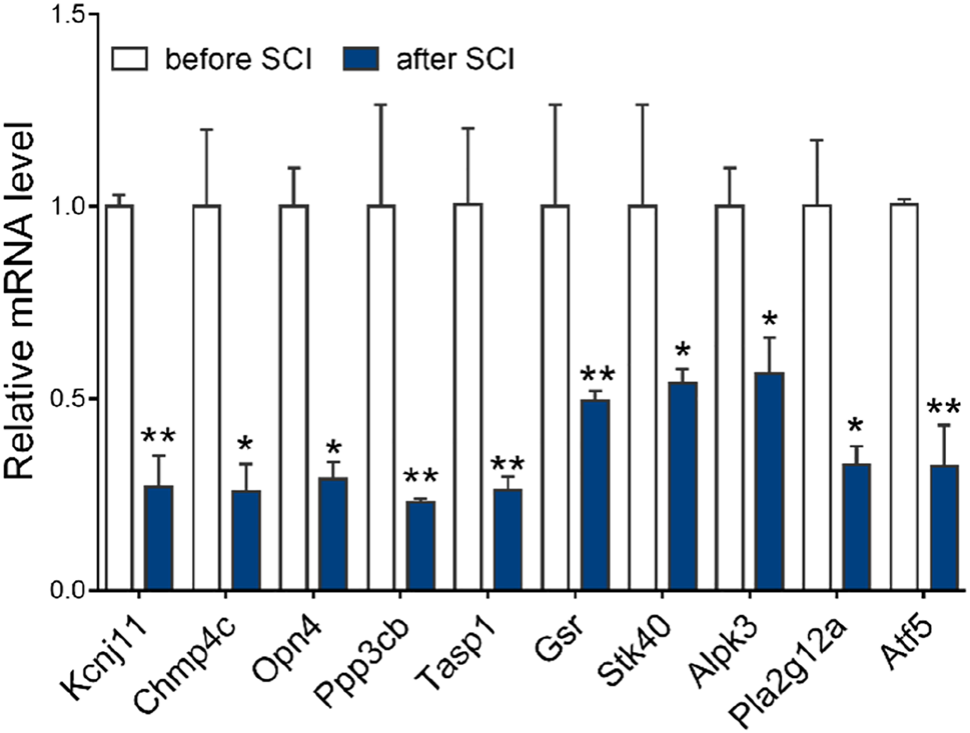
Verification using the mRNA expression. *Kcnj11*, *Chmp4c*, *Opn4*, *Ppp3cb*, *Tasp1*, *Gsr*, *Stk40*, *Alpk3*, *Pla2g12a*, and *Atf5* expression in before SCI (n = 6) and after SCI (n = 6) were evaluated by qPCR and normalised against the corresponding glyceraldehyde-3-phosphate dehydrogenase (*GAPDH*) expression. An asterisk represents *p* < 0.05 and two asterisks indicate *p* < 0.01 when compared with the before SCI group.

To verify the reliability of the microarray results, we used qRT-PCR to detect the expression levels of 10 genes (*Kcnj11*, *Chmp4c*, *Opn4*, *Ppp3cb*, *Tasp1*, *Gsr*, *Stk40*, *Alpk3*, *Pla2g12a*, and *Atf5*), and the results were consistent with the microarray data. These 10 genes were selected because they showed the greatest significance in the microarray results. The difference in mRNA expression of these genes between the before SCI group and the after SCI group was significant (*P* < 0.05) (Fig. 5).

## Discussion

Currently, no clear consensus exists regarding the classification of muscle atrophy after SCI. Some reports have suggested that muscle atrophy after SCI be classified as disuse atrophy or denervation atrophy^15–18^. However, muscle atrophy after SCI differs from these types of muscle atrophy. Disuse atrophy is caused by a significant loss of muscle mass and strength due to limb immobilisation, long-term bed rest, lack of physical activity, or space flight^19^. Denervation atrophy is usually induced in peripheral neurotomy models, where the central nervous system is not damaged. The difference between muscle loss caused by SCI and the other types of atrophy is that lower motor neurons remain intact in SCI and upper motor neurons cannot transmit information to the lower motor neurons. Previous studies on muscle atrophy have focused on animal models of disuse atrophy and denervation atrophy; muscle atrophy after SCI is usually classified as either one or a combination of these muscle atrophy models. However, the pathogenesis of muscle atrophy after SCI involves multiple factors, including signal transduction, immunity, electrical conduction, stimulation, and metabolism^20,21^. This complex situation cannot be attributed to a single molecular mechanism or simple combinations. The gene profile data in the present study employed a model of moderate SCI to simulate the clinical phenomenon of muscle atrophy after SCI.

Chen et al.'s study^22^ used key genes in the co-expression network to classify all SCI samples in GSE45550, including 4 groups (before SCI, 3, 8 and 14 days following SCI). It is believed that the expression levels of the first six genes (CCNB2, CCNB1, CKS2, COL5A1, KIF20A, and RACGAP1) can be used as potential marker genes for different SCI subtypes. Niu et al.'s study^23^ used bioinformatics analysis to explore differentially expressed genes associated with the acute and chronic stages of SCI, starting from 1 day to 6 months after SCI, including data sets (GSE45006, GSE93249, and GSE45550). Similarly, Zhang et al.'s research^24^ conducted bioinformatics analysis on the data set GSE45550 (including 4 groups: before SCI, 3, 8 and 14 days following SCI) to identify key genes or important signal pathways related to SCI. This study focuses on a part of the data set GSE45550 (two groups: before SCI and 14 days following SCI), and compares the differentially expressed genes of the two groups. The results show that the same disease may have very different results and potential differences at different stages. This reminds us that the same patient needs different treatment adjustments at different stages of the disease.

A previous microarray analysis by Urso et al.^25^ was used to describe new gene expression involved in signalling pathways that regulate the loss of muscle proteins or increased response to atrophy stimuli in the first few days after SCI. Microarray analysis revealed that metallothionein activity (*MT2A*, *MT1A*, *MT1E*, *MT1F*, *MT1G*, *MT1H*, *MT1M*, *MT1R*, and *MT1X*) and protease inhibition activity (secretory leukocyte protease inhibitor) increased significantly at 2 and 5 days after SCI^25^. Our microarray analysis revealed that the 10 genes with the most significant differential expression at 14 days after SCI were *Kcnj11*, *Chmp4c*, *Opn4*, *Kcnj11*, *Tasp1*, *Gsr*, *Stk40*, *Alpk3*, *Pla2g12a*, and *Atf5*. This indicated that the changes in mRNA expression of muscle atrophy at different times after SCI were different. Shen et al*.*^26^ first proposed that innervation atrophy can be divided into four different transcription stages: oxidative stress stage (0–12 h), inflammation stage (24 h), atrophy stage (3–7 days), and atrophic fibrosis stage (14–28 days). Whether muscle atrophy after SCI involves similar stages remains to be further studied.

The GO analysis showed that the primary biological processes of differential genes were ‘response to drug’, ‘fatty acid beta-oxidation’, ‘response to hypoxia’, ‘biological_process’, ‘heart development’, ‘positive regulation of transcription, DNA-dependent’, ‘response to glucose stimulus’, ‘response to nutrient’, ‘positive regulation of transcription from RNA polymerase II promoter’, and ‘positive regulation of apoptotic process’. These results showed that the pathogenesis of muscle atrophy after SCI is the result of mutual gene and network regulation of multiple factors and genes that directly or indirectly lead to the development and progression of muscle atrophy. Among them, ‘positive regulation of apoptotic process’ warrants further study because skeletal muscle atrophy is closely associated with apoptosis, but the specific mechanisms of action are not well understood^27^. Studies^28,29^ have shown that atrophy and death of skeletal muscle cells lead to sarcopenia, which is a disorder associated with normal ageing. It is estimated that 30–40% of skeletal muscle fibres are usually lost by the age of 80 years^30^. Although the mechanisms of this loss are not well understood, it is possible that apoptosis is involved. Mitochondrial dysfunction and sarcoplasmic reticulum stress that occur with increasing age may be possible factors that induce apoptosis. Therefore, the mitochondria and sarcoplasmic reticulum may be important organelles in skeletal muscle cells responsible for signal transduction associated with apoptosis. The activation of apoptosis may be a part of the reason for the initiation of muscle protein degradation, loss of muscle nuclei associated with local atrophy, and death of muscle cells. Exercise training and calorie restriction are two interventions known to enhance skeletal muscle function^30^.

In the present study, DEGs were primarily enriched in the pathways ‘metabolic pathways’, ‘PPAR signalling pathway’, ‘peroxisome’, ‘fatty acid degradation’, ‘fatty acid elongation’, ‘glutathione metabolism’, ‘propanoate metabolism’, ‘valine, leucine and isoleucine degradation’, ‘adipocytokine signalling pathway’, and ‘glyoxylate and dicarboxylate metabolism’, of which the ‘fatty acid degradation’ and ‘fatty acid elongation’ pathways are consistent with the major pathways (fatty acid metabolism) analysed in the study of muscle atrophy after SCI by Dirks et al^30^*.* These pathways are believed to be the key players in fatty acid metabolism (Lpl and Fabp3) and have proved to be the major sensors of SCI-induced inactivity and reloading with training. Pathway network analysis showed that ‘citrate cycle (TCA cycle)’, ‘pyruvate metabolism’, ‘MAPK signalling pathway’, ‘fatty acid degradation’, ‘propanoate metabolism’, ‘apoptosis’, ‘focal adhesion’, ‘synthesis and degradation of ketone bodies’, ‘Wnt signalling pathway’, and ‘pathways in cancer’ were the 10 pathways with the highest degree, indicating that these signalling pathways may play the most important roles in muscle atrophy after SCI. Among them, the focal adhesion pathway warrants further attention because some studies^31^ have reported that it may play an important role in amyotrophic lateral sclerosis. In addition, studies^32^ have reported that the mechanisms and strategies to counter muscle atrophy also include the focal adhesion pathway. The primary sensors for the changes in muscle activity (and load) are the integrin complex and the dystrophin-associated protein complex. Neither of these complexes has inherent signalling function, but they appear to mediate focal adhesion through focal adhesion kinase (FAK)^33,34^, a targeted kinase that indirectly provides nutrients through many different pathways, thereby leading to the activation of growth pathways and preventing apoptosis. Total FAK and its activity were reduced in atrophic soleus muscle, and other proteins associated with focal adhesion complexes were also downregulated^35^.

In the gene signal network analysis, *Acat1*, *Acadvl*, *Acaa2*, *Hadhb*, *Acss1*, *Oxct1*, *Hadha*, *Hadh*, *Acaca*, and *Cpt1b* may play key roles in the development and progression of muscle atrophy after SCI because they had the highest betweenness values (higher betweenness indicates more connections with a gene, suggesting that the gene may be more important). The results also showed the relationship between these 10 genes, providing new avenues for the prevention and treatment of muscle atrophy after SCI. Among them, the betweenness value of *Acadvl* was the highest. *Acadvl* is involved in the fatty acid degradation pathway^36^ and the fatty acid beta-oxidation biological process^37^. However, the fatty acid degradation pathway was screened from the pathway analysis and pathway network analysis, and fatty acid beta-oxidation was also screened from the GO analysis. These showed the interdependency of the results; they also suggest that *Acadvl* may be a key regulatory gene in the development of muscle atrophy after SCI, warranting further study. ACADVL is responsible for the production of very long-chain acyl-CoA dehydrogenase, an enzyme that plays a key role in the mitochondria. It is essential for fatty acid oxidation, a multi-stage process that metabolises fat into energy^38^. There is a close relationship between abnormalities associated with neurodegenerative diseases such as Alzheimer’s disease and brain fatty acid metabolism. The pathology associated with Alzheimer’s disease inhibits the homeostasis and regeneration of neural stem cells by interfering with fatty acid metabolism^39,40^.

The sample size of the data in the present study was small and the samples were selected from a platform, which may lead to some degree of false negatives. Further experimental studies and a larger sample size are needed to confirm the results of the present study. A key limitation of the original study design is that, while the SCI group underwent a laminectomy and injury, the control group did not undergo ‘sham’ surgery. Thus, the study was not controlled for surgery effects.

In summary, the genetic profile data of atrophic muscle tissue before SCI and at 14 days after moderate SCI was downloaded from the Gene Expression Omnibus to identify genes differentially expressed between the two conditions. In addition, bioinformatic analyses were performed to gain new insights into the molecular mechanisms of muscle atrophy in this model. It is still not clear whether muscle atrophy 14 days after SCI in this model properly represents muscle atrophy in the atrophic fibrosis stage. Nevertheless, our current understanding of the mechanisms underlying muscle atrophy after SCI is undoubtedly expanded, and several potential targets for the prevention and treatment of this condition were also revealed.

## Materials and methods

### Gene profile data

The keywords ‘SCI’ and ‘muscle atrophy’ were used to search the Gene Expression Omnibus in the National Centre for Biotechnology Information (NCBI) database (http://www.ncbi.nlm.nih.gov/geo/) and the GSE45550 gene profile dataset was obtained. These data represented a control group (before SCI, n = 6) and an experimental group (14 days after SCI, n = 6). The sample tissue was soleus muscle tissue of Sprague–Dawley rats. The platform was GPL1355 [Rat230_2] Affymetrix Rat Genome 230 2.0 Array. The SCI model was established by anaesthetising the rat and dropping a 10 g cylinder from a height of 25 mm at the level of T7–T9 after a dorsal laminectomy, affecting the spinal cord at T8 to cause injury^7^. All procedures were performed in accordance with the US Government Principle for the Utilization and Care of Vertebrate Animals and were approved by the Institutional Animal Care and Use Committee at the University of Florida. The study was carried out in compliance with the ARRIVE guidelines (https://arriveguidelines.org).

### Design of analysis process

**Figure 6:**
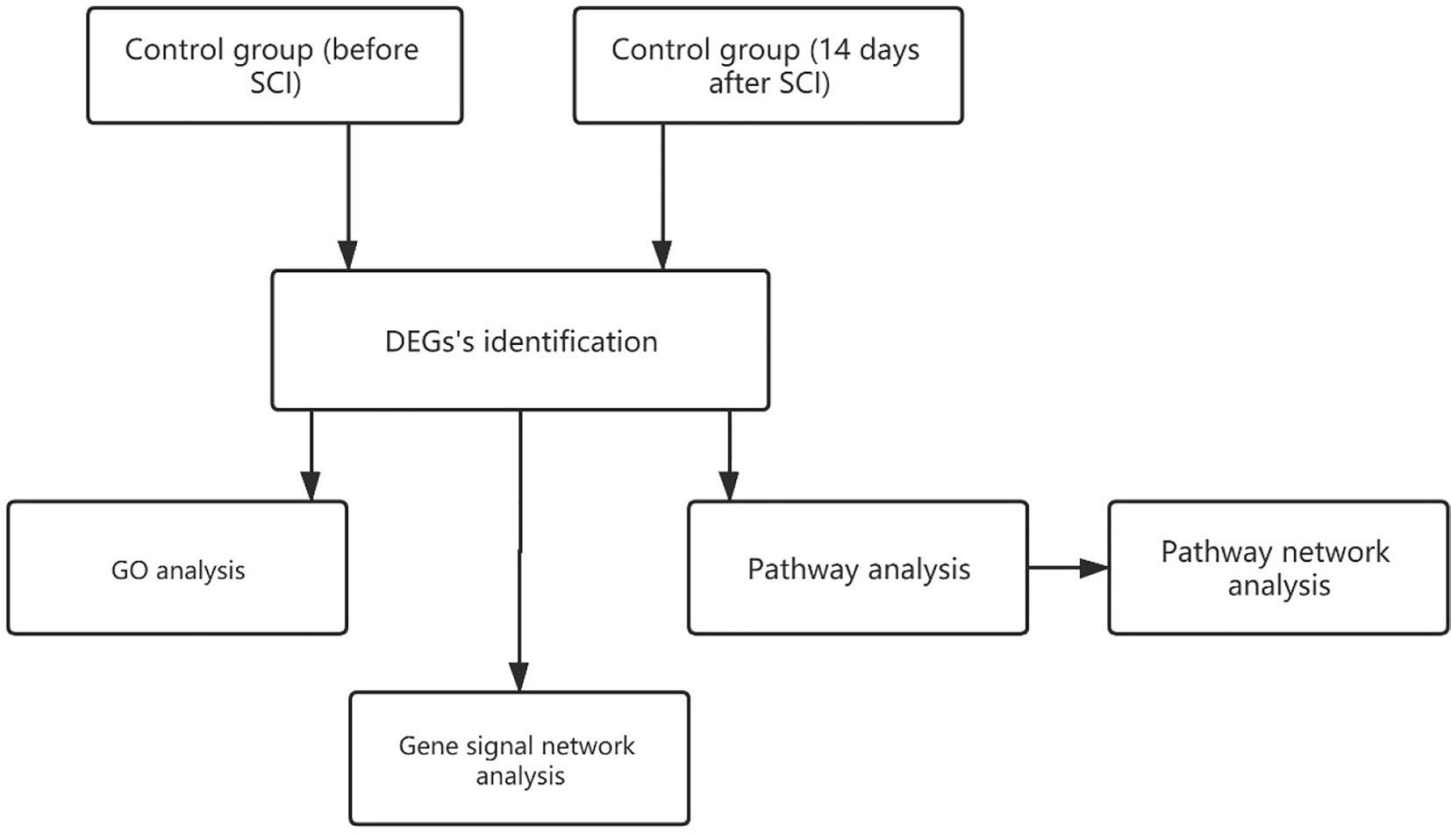
Flowchart of the bioinformatic analysis in the present study. SCI, spinal cord injury. DEGs, differentially expressed genes. n = 6/group.

The GSE45550 gene profile dataset was input into the Gene-Cloud of Biotechnology Information (GCBI) analysis platform (https://www.gcbi.com.cn/gclib/html/index) to perform all the bioinformatics analyses in this study. The details of this process are shown as a flowchart (Fig. 6). In the GCBI, complex biological information analysis can be completed by dragging the ‘Move’ module and clicking ‘Operation’. Several bioinformatic studies have used the GCBI to discover new genes and molecular mechanisms in diseases and improve the understanding of the underlying molecular mechanisms.

### Identification of DEGs

For DEG identification, significance analysis of microarrays (SAM)^41^ was used to screen for genes in previously designated groups with significant differential expression. First, SAM was used to calculate a statistic (d score) for each gene to measure the degree of correlation between the gene expression level under the corresponding grouping and the designated group, and then simulated the distribution of these statistics randomly (randomly replaced samples) through a series of 1,000 replacements. The statistic value (d Score) was calculated for each gene di = ri/(si + s0), where ri reflects the difference in the average level among different groups and si reflects the variation in sample population. Considering the influence of the variance of low-abundance genes and the correlation between genes, the statistical calculations, and the methods for evaluating statistical significance were adjusted using appropriate methods to obtain the significance level for each gene. Finally, to adjust for the *p* values of multiple experiments, a *q* value was calculated by constructing the discretised rejection region to control the FDR^42,43^. Differences with q < 0.05 with fold change > 1.5 or <  − 1.5 were considered statistically significant.

### GO analysis

The GO database is a cross-species, comprehensive, and descriptive database established by the Gene Ontology Consortium. In the GO analysis, the DEG between the groups was used to perform gene function annotation based on the GO database to obtain all the functions in which the gene is involved. Next, Fisher’s exact test and multiple comparison tests were used to calculate the significance level (*p* value) and FDR in order to screen the significant functions of each differentially expressed gene. The enrichment results with FDR < 0.05 were considered statistically significant.

### Pathway analysis

The Kyoto Encyclopaedia of Genes and Genomes (KEGG) is a database that systematically analyses the relationship between genes (and their coded products), gene functions, and genomic information, and allows the study of genes and their expression as an entire network. Based on the KEGG database analysis, Fisher’s exact test was used for the differential genes to perform significance analysis of the pathways in which the target gene is involved in order to screen the significant pathways of each differential gene. Pathways with FDR < 0.05 were considered statistically significant.

### Pathway network analysis

Pathway network analysis allows comprehensive, systematic analysis of the signal transduction relationships among significant pathways discovered from pathway analysis and intuitive discovery of the synergistic effects of major pathways when the sample changes, thereby providing a systematic understanding of the nature of the changes in sample traits. The pathway network analysis was used to construct an interaction network between pathways based on the interaction relationships in the KEGG database. The network was determined based only on the significant pathways from the pathway analysis. The calculation and description of network attribute: network N is denoted as ${\text{N}}: = \left( {{\text{V}},{\text{E}}} \right)$, where V is the node set whose totality is n, and E is the edge set whose totality is m. The corresponding adjacent matrix is noted by $A: = \left[ {a_{ij} } \right],i,j \in V$, where ${\text{a}}_{{{\text{ij}}}} = 1$ if i connected to j, or else ${\text{a}}_{{{\text{ij}}}}$ = 0. The degree of node i:${\text{D}}\left( {\text{i}} \right): = \sum\nolimits_{j = 1}^{n} {a_{ij} }$. Indegree values: the number of upstream pathways of a certain pathway; outdegree values: the number of downstream pathways of a certain pathway; degree values: the number of upstream and downstream pathways of a certain pathway; core pathways: degree values ≥ 1.

### Gene signalling network analysis

The interaction between genes and gene products in the KEGG database and the interaction between each gene and other genes obtained from searching the database allow the interaction between the target gene clusters to be comprehensively discovered and positioned as upstream and downstream proteins. Next, an interaction network between genes was constructed. The network based on the DEGs. The betweenness of i:$\mathrm{B}\left(\mathrm{i}\right)=\sum_{s\ne i\ne t}\frac{{\sigma }_{st}(i)}{{\sigma }_{st}}$, where ${\sigma }_{st}$ denotes the total number of the shortest path from node s to node t, ${\sigma }_{st}(i)$ denotes the total number of the shortest paths through node i from node s to node t. Betweenness values: signal transmission mediation centre, if the value is larger, the mediation ability of a certain gene in signal transmission is stronger. Indegree values: the number of upstream genes of a certain gene; outdegree values: the number of downstream genes of a certain gene; degree values: the number of upstream and downstream genes of a certain gene; hub genes: degree values ≥ 1.

### Validation of the mRNAs using qRT-PCR

Twelve adult female Spragdorley rats (16 weeks of age, 260–280 g at the beginning of the study, Slaccas Laboratory, Shanghai, China) were reared at a temperature of 22 ± 1 °C, humidity of 50% ± 10%, and light: dark cycle of 12:12, and provided a sufficient food and water. Animals were sacrificed: a control group (before SCI, n = 6) and an experimental group (14 days after SCI, n = 6). The SCI model^7^ was established by anaesthetising the rat and dropping a 10 g cylinder from a height of 25 mm at the level of T7–T9 after a dorsal laminectomy, affecting the spinal cord at T8 to cause injury. The animals were deeply anaesthetised with a combination of ketamine (90 mg/kg body weight) and xylazine (8 mg/kg body weight). The sample tissue was soleus muscle tissue. Beyozol (Beyotime Bio, Inc., China) was used to extract total RNA from the frozen tissues. The BeyoRT II cDNA Synthesis Kit (Beyotime Bio, Inc., China) was used to reverse transcribe RNA into cDNA. Thereafter, 2 µg of cDNA was tested in each reaction using the BeyoFast SYBR Green One-Step qRT-PCR Kit (Beyotime Bio, Inc., China) in the Applied Biosystems Real-Time PCR System (Applied Biosystems; Thermo Fisher Scientific). The 2^−ΔΔCt^ method was adopted to calculate the expression of genes relative to the housekeeping gene *GAPDH*. Table 6 shows the primers applied to qRT-PCR. The Ethics Committee of Hainan General Hospital of China approved the experiment (Approval No: Med-Eth-Re [2020] 12).

**Table 6:** Primers used in qRT-PCR.

Gene	Forward sequence	Reverse sequence
Kcnj11	TGCGTCACAAGCATCCACTCCT	GGACATTCCTCTGTCACCATGC
Chmp4c	GCAGGACATTGCTGACCAGCAA	CAAGTTCCTCCAACTCTGCCATG
Opn4	CTACTCCACTGTGGCTCTGGTG	TTGTGGATGGCAGAAGCCTTGG
Ppp3cb	GACAGAAGGTGAAGACCAGTTTG	TCAGCACGCTTTCACTCTCCTC
Tasp1	TGGAGAAGGAGCCTACAGATGG	CTGTTTCCACCCTTTCTGCCAG
Gsr	GTTTACCGCTCCACACATCCTG	GCTGAAAGAAGCCATCACTGGTG
Stk40	AGGCTCTCAGTGCCATCATTGC	CTCGTATTGGGAGCACTCCTCT
Alpk3	CAAACGAGCCACAAGACTCCAG	CCTTGATTTCCAGAGCTGTCGTG
Pla2g12a	GCAAGAACGACTGTGACGAGGA	GATGACGCTGTCAAAGAGGAGC
Atf5	GCTCGTAGACTATGGGAAACTCC	CAGTCATCCAATCAGAGAAGCCG
GAPDH	CATCACTGCCACCCAGAAGACTG	ATGCCAGTGAGCTTCCCGTTCAG

## Supplementary Information

Supplementary Information 1.

Supplementary Information 2.

Supplementary Information 3.

## Data Availability

Authors declare the availability of all data.
