# Supplementary material for: Bioinformatic analysis of the gene expression profile in muscle atrophy after spinal cord injury
Source: Sci Rep. 2021 Nov 9;11:21903. doi: 10.1038/s41598-021-01302-6 (PMC8578571; doi:10.1038/s41598-021-01302-6)
Supplement: Supplementary file 2 — Supplementary Information 2. [file 41598_2021_1302_MOESM2_ESM.docx]

# Method Description of GO and Pathway

**One. The significant analysis of Gene Ontology (GO)**

GO-Analysis is the analysis for selecting significant GO. Because the hierarchical relation of the terms in GO, the number of gene in single GO term varies in a large range, usually one to several hundred genes. GCBI used Fisher exact test for GO analysis, which would be represented in the following 2*2 contingency table:

|  | Difference Gene | Non-difference Gene | Total |
| --- | --- | --- | --- |
| Genes in GO | $n_{f}$ | $n-n_{f}$ | $n$ |
| Genes out of GO | $N_{f}-n_{f}$ | $(N-N_{f})-(n-n_{f})$ | $N-n$ |
| Total | $N_{f}$ | $N-N_{f}$ | $N$ |

$n_{f}$：the number of difference genes in GO；$N_{f}$：the number of difference genes；$n$：the number of genes in GO；$N$: the total number of genes in the annotation system. Under given marginal frequency (i.e. the gene number is constant in GO), the elements in the crosstab obey the hypergeometry distribution, then we would calculate the p-value for Fisher exact test. That is to say that we would have the p-value of Fisher exact test by solve the cumulative hypergeometry distribution values with two tails. For $2n_{f}\leq n$, the formula of p-value as follows:

$p_{F}\left( n_{f},n,N_{f},N \right)=2*p_{h}\left( X\leq n_{f} \right)=2*\sum_{x=1}^{n_{f}} \frac{\left( \begin{matrix} n \\ x \end{matrix} \right)\left( \begin{matrix} N-n \\ N_{f}-x \end{matrix} \right)}{\left( \begin{matrix} N \\ N_{f} \end{matrix} \right)}$.

In order to control the whole probability of the type one error in multiple hypothesis test, we use the Benjamini-Hochberg step-up method to control FDR (see reference [1] for details), then we obtain the statistical significant GO. Besides, we would calculate the enrichment score to access the enrichment level for per GO. The calculate formula as follows：

$R_{e}=\frac{{n_{f}}/n}{{N_{f}}/N}$.

[1] Y. Benjamini, Y. Hochberg (1995). Controlling the false discovery rate: a practical and powerful approach to multiple testing. Journal of the Royal Statistical Society, Series B 57 (1): 289–300.

**Two. The significant analysis of KEGG Pathway**

Pathway-Analysis is the significant analysis of Pathway which these difference genes participated. For the whole biochemical process include metabolism, signal transmission and cell cycle et al information, which contained in the Pathway, single Pathway usually contains hundreds of genes. GCBI mainly utilize the Fisher exact test for significant analysis of Pathway, see the following contingency table:

|  | Difference Gene | Non-difference Gene | Total |
| --- | --- | --- | --- |
| Genes in GO | $n_{f}$ | $n-n_{f}$ | $n$ |
| Genes out of GO | $N_{f}-n_{f}$ | $(N-N_{f})-(n-n_{f})$ | $N-n$ |
| Total | $N_{f}$ | $N-N_{f}$ | $N$ |

$n_{f}$：the number of difference genes in Pathway；$N_{f}$：the number of difference genes；$n$：the number of genes in Pathway；$N$: the total number of genes in the annotation system. Under given marginal frequency (i.e. the gene number is constant in Pathway), the elements in the crosstab obey the hypergeometry distribution, and then we would calculate the p-value for Fisher exact test. That is to say that we would have the p-value of Fisher exact test by solve the cumulative hypergeometry distribution values with two tails. For $2n_{f}\leq n$, the formula of p-value as follows:

$p_{F}\left( n_{f},n,N_{f},N \right)=2*p_{h}\left( X\leq n_{f} \right)=2*\sum_{x=1}^{n_{f}} \frac{\left( \begin{matrix} n \\ x \end{matrix} \right)\left( \begin{matrix} N-n \\ N_{f}-x \end{matrix} \right)}{\left( \begin{matrix} N \\ N_{f} \end{matrix} \right)}$.

In order to control the whole probability of the type one error in multiple hypothesis test, we use the Benjamini-Hochberg step-up method to control FDR (see reference [1] for details), then we obtain the statistical significant Pathway. Besides, we would calculate the enrichment score to access the enrichment level for per Pathway. The calculate formula as follows：

$R_{e}=\frac{{n_{f}}/n}{{N_{f}}/N}$.

[1] Y. Benjamini, Y. Hochberg (1995). Controlling the false discovery rate: a practical and powerful approach to multiple testing. Journal of the Royal Statistical Society, Series B 57 (1): 289–300.


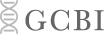


GCBI copyright, GCBI all rights reserved. Without the written authorization of GCBI, any organization or individual shall not copy this document, copy it, lease it, burn it on CDR, transfer, compile, modify and save the public information system (such as Internet, BBS), and change to a different language version, or any other matters in violation of copyright laws and international copyright conventions.

Copyright© 2014-2015 GCBI. All rights reserved.
