# Supplementary material for: Bioinformatic analysis of the gene expression profile in muscle atrophy after spinal cord injury
Source: Sci Rep. 2021 Nov 9;11:21903. doi: 10.1038/s41598-021-01302-6 (PMC8578571; doi:10.1038/s41598-021-01302-6)
Supplement: Supplementary file 3 — Supplementary Information 3. [file 41598_2021_1302_MOESM3_ESM.docx]

# Method Description of Network Analysis

A biology network reflects the relation among the genes or the relation between genes and GO or pathway et al. By analyzing this biology network, we could achieve some assistant “venation” which effect the experiment importantly, and systematically “excavate” the real clue in the whole complex scope chain. This document contained the details of six category analysis method, see the corresponding title for reference.

**Introduction to the Analysis Methodology of GeneSignalNetwork**

GeneSignalNetwork deconstructs the KEGG database, which breakthrough the limit of acquiring the interactions of between genes in single pathway. Therefore, GeneSignalNetwork can obtain some protein's upstream or downstream proteins through the whole KEGG-Pathway database. The methods of construction are listing below.

1. Upstream and downstream genes should meet the filter conditions simultaneously, when searching them in databases.

2. Drawing lines between genes according to the upstream and downstream interactions between genes. Genes are denoted by dots and interactions are denoted by lines.

2.1 binding/association –line marked as 'b': a complex combined by two regulatory protein; non-directional impact; no arrow on the line.

2.2 Phosphorylation –line marked as 'pho': Protein A transfer the phosphate group of ATP or GTP to protein B, to active the function of another protein. The function has direction. It means that the interaction between protein A and protein B linked with arrow line.

2.3 Ubiquitination –line marked as 'u': The process of the specific modification from protein A to its target protein B. The interaction between 2 proteins has direction, so it's an arrow line.

2.4 Expression –line marked as 'exp': The transcription of a DNA activated by protein A, in order to increase the expression. The interaction has its action, and line is an arrow line. It means that the activation of a gene's transcription expression regulation from protein A to another gene has its direction, and the line has an arrow.

2.5 Activation –line marked as 'a': Through the interaction between two proteins, one protein activate the function of another. The function has its direction. It means that the interaction from one protein activating another has its direction, and the line has an arrow.

2.6 Inhibition –line marked as 'inh': Through the interaction between 2 proteins, one protein inhibits the function of another. It means that the interaction of inhibition from protein A to another protein has its direction, and the line is a flat note.

2.7 Indirect –line marked as 'ind': Protein A can have effect with protein B, but the implementation of the interaction can be done only through other protein or signal path as an intermediation. The function has its direction. It means that the indirect effect from protein A to another protein has direction, and the line of function has arrow.

2.8 compound –line marked as 'c': As two nearby metabolic enzymes, protein A and protein B metabolize the same compound. The interaction has no direction, so the line between has no arrows.

GCBI will exclude genes which has discordant regulation of up or down in mRNA expression profile database. Then, for those genes has regulation of up or down, negative association will be taken by default in miRNA and mRNA expression profile DB. Negative association won't be taken for multi-group differentiation result. Instead, intersection will be taken as a result. Finally, the computation of Network Diagram attribution are listed in Appendix III.

**Introduction to the Analysis Methodology of PathwayRelationNetwork**

Pathway here is referenced to signal pathway. According to the method of graph theory, pathway is taken as the subject unit. The interaction in KEGG DB are used to construct the interaction network between pathways. The overall and systematically analysis of the relationship between marked pathways which discovered by Pathway-Analysis, can help to find out the synergistic effect model of important pathway when sample changes. And it can help to understand the essence of the change of samples. Pathway Relation Network can help us find out the pathway who has regulating effect on the top stream, and on the lowest stream at the same time. Via the comprehension of the relationship between pathways, we can have a deeper acquaintance about signal pathways.

GCBI will show the Gene Feature included in marked pathway through the Field – Pathway Feature. Other computations of Network Diagram attribution are listed in Appendix III.

## Appendix

I. Introduction to Weighted-Network Analysis

Weighted-network is a simple complete network. Every node denote a gene, the weight of every edge is used to measure the strength of the interaction between two genes. Let $i, j \in V$, and using $s_{ij}=|cor\left( i, j \right)|$ to denote the absolute value between gene i and j. Then, the weight between i and j can be valued as:

$a_{ij}=s_{ij}^{\beta}$，

where, beta is an arbitrary positive integer. All the weights consist into a symmetric matrix ($a_{ij}$) (where its diagonal elements are all 0). Weighted-network adjacent matrix can be viewed as the generalization of network adjacent matrix. Denoted as the adjacent matrix of weighted-network (V, E). Naturally, we call the sum of a row (or a column) of the matrix as the connection degree of relating node:

$k_{i}=\sum_{j} a_{ij}$.

One of the most important purpose of co-expression network analysis, is to find out the subset of the nodes which have closer relationships, which is denoted as a module. So, we should define the dissimilarity between two nodes. Practice shows that dissimilarity defined by topological overlap of two nodes has a better effect:

$\omega_{ij}=\frac{\sum_{u} a_{iu}a_{uj}+a_{ij}}{\min\left( k_{i}, k_{j} \right)+1-a_{ij}}$,

where $k_{i}$ is the connection degree of node i. In no-weighted-network, $\omega_{ij}=1$ if and only if all the adjacent nodes of the node with less connection degree in nodes i, j must be the adjacent point of another node, and node i, j must be adjacent. While $\omega_{ij}=0$ if and only if there's no common adjacent point of node i, j and i, j is not adjacent. If it's a weighted-network, note that:

$\sum_{u} a_{iu}a_{uj}\leq min(\sum_{u\neq j} a_{iu}, \sum_{u\neq i} a_{uj})$,

then

$\sum_{u} a_{iu}a_{uj}\leq\min\left( k_{i}, k_{j} \right)-a_{ij}$,

so $0\leq\omega_{ij}\leq1$. Therefore, the idea of topological coverage can naturally be generalized to weighted-network. With topological coverage, the dissimilarity between node i, j can be defined as:

$d_{ij}^{\omega}=1-\omega_{ij}$.

After calculating the dissimilarity between genes, we can apply cluster analysis to it, in order to get the module which the network has. Practice shows that many biological co-expression network will satisfy the property of scale-free: that is the connection degree of the nodes k satisfies the power law distribution:

$p(k)\sim k^{-\gamma}$,

Scale-free networks usually have several modules. It means that there's several subset nodes whose nodes has low dissimilarity, while dissimilarity between modules are relatively high and the connection degree between modules are relevantly low. Besides, intuitively, scale-free network is highly inhomogeneous: there are few nodes with high connection degree, and these nodes always relates to key genes. These relating genes usually perform some important biological functions. We call these genes the hub. The main purpose of gene co-expression analysis is to find the hubs. According to the consideration of the definition of scale-free network and biological functions, if the weighted-network of a group of gene satisfies the properties of scale measure, it must satisfy the following standards: First, log (p (k)) and log (k) have a linear relation and the slope should be near -1. Second, average connection degree should not be too low.

II. The description of weighted-network diagram’s presentation

Since weighted-network graph is a complete graph and the quantity of the analysis gene is huge, if the demonstration of complete graph cannot stress the key point, we should extrude the genes with high connection degrees. In order to show the connection degree of weighted-network honestly, hard threshold is not a solution. Besides, for the clarity of the demonstration, the average connection degree should be near 1.

If the condition of the scale-free network is meet, the method of generating a network is as follows: Calculate the connection degrees of every node for the adjacent matrix, then round to integer and we get an integer connection degree for every node. Divide sum of these integers by the node number, then we will get a reasonable average connection degree w for this adjacent matrix. If the connection degree of some node is d, find out the corresponding row in the adjacent matrix and take the d-th largest weight in the row as a candidate threshold. A series of candidate threshold will be found after this process, and we sort it. For every candidate threshold, we can get a new 0-1 adjacent matrix which makes the ones in the new matrix map to the position of the origin matrix which has a larger weight than this threshold.

If the new matrix contains a row (or column) with all zeros elements, and the corresponding node integer connection degree d is not less than 1, then change the value in this row (or column) in order to make it contains d of ones mapping to the position of first d largest values in the origin adjacent matrix. So the average connection degree of the scale-free diagram corresponding to this new 0-1 adjacent matrix can be calculated using the sum of the new matrix divided by the gene number. In order to make the average connection degree close to the w-value we calculated before, we use dual searching a new candidate threshold. We denote this value as $k_{w}$. If $k_{w}$ is larger than 1, we increase the value of beta by some step, and then calculate $k_{w}$ in the same way until $k_{w}$ is less than 1. So that we will get a series of candidate beta. Again using the dual searching method for the most reasonable beta will make the corresponding $k_{w}$ approaching to 1. Then, the 0-1 adjacent matrix with this $k_{w}$ is the matrix that we demonstrate finally.

III. The calculation and description of network attribute.

Network N is denoted by $N:=(V,E)$, where V is the node set whose totality is n, and E is the edge set whose totality is m. the corresponding adjacent matrix is noted by $A:={[a}_{ij}], i,j\in V$, where $a_{ij}=1$ if i connected to j, $a_{ij}$ = 0 else.

The degree of node i:$D(i):=\sum_{j=1}^{n} a_{ij}$.

The betweenness of i:$B\left( i \right)=\sum_{s\neq i\neq t} \frac{\sigma_{st}(i)}{\sigma_{st}}$, where $\sigma_{st}$ denotes the total number of the shortest path from node s to node t, $\sigma_{st}(i)$ denotes the total number of the shortest path which through node i from node s to node t.

The clustering co-efficient of no-direction node i: $B\left( i \right)=\frac{\lambda\left( i \right)}{\tau\left( i \right)}$, where $\lambda\left( i \right)$ denotes the total number of nodes who includes node i and has a subgraph containing 3 nodes and 3 edges, and $\tau\left( i \right)$ denotes the total number of nodes who includes node i and has a subgraph containing 3 nodes and 2 edges which connected to node i.


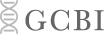


GCBI copyright, GCBI all rights reserved. Without the written authorization of GCBI, any organization or individual shall not copy this document, copy it, lease it, burn it on CDR, transfer, compile, modify and save the public information system (such as Internet, BBS), and change to a different language version, or any other matters in violation of copyright laws and international copyright conventions.

Copyright© 2014-2015 GCBI. All rights reserved.
